# Identifying protein complexes based on density and modularity in protein-protein interaction network

**DOI:** 10.1186/1752-0509-7-S4-S12

**Published:** 2013-10-23

**Authors:** Jun Ren, Jianxin Wang, Min Li, Lusheng Wang

**Affiliations:** 1 School of Information Science and Engineering, Central South University, Changsha 410083, Hunan, P. R. China; 2 College of Information Science and Technology, Hunan Agricultural University, Changsha 410128, Hunan, P.R. China; 3 Department of Computer Science, City University of HongKong, 83 Tat Chee Avenue, Kowloon, Hong Kong, P.R. China

## Abstract

**Background:**

Identifying protein complexes is crucial to understanding principles of cellular organization and functional mechanisms. As many evidences have indicated that the subgraphs with high density or with high modularity in PPI network usually correspond to protein complexes, protein complexes detection methods based on PPI network focused on subgraph's density or its modularity in PPI network. However, dense subgraphs may have low modularity and subgraph with high modularity may have low density, which results that protein complexes may be subgraphs with low modularity or with low density in the PPI network. As the density-based methods are difficult to mine protein complexes with low density, and the modularity-based methods are difficult to mine protein complexes with low modularity, both two methods have limitation for identifying protein complexes with various density and modularity.

**Results:**

To identify protein complexes with various density and modularity, including those have low density but high modularity and those have low modularity but high density, we define a novel subgraph's fitness, *f_ρ_*, as *f_ρ_*= (*density*)^ρ^*(*modularity*)^1-*ρ*^, and propose a novel algorithm, named LF_PIN, to identify protein complexes by expanding seed edges to subgraphs with the local maximum fitness value. Experimental results of LF-PIN in *S.cerevisiae *show that compared with the results of fitness equal to density (ρ = 1) or equal to modularity (ρ = 0), the LF-PIN identifies known protein complexes more effectively when the fitness value is decided by both density and modularity (0<ρ<1). Compared with the results of seven competing protein complex detection methods (CMC, Core-Attachment, CPM, DPClus, HC-PIN, MCL, and NFC) in *S.cerevisiae *and *E.coli*, LF-PIN outperforms other seven methods in terms of matching with known complexes and functional enrichment. Moreover, LF-PIN has better performance in identifying protein complexes with low density or with low modularity.

**Conclusions:**

By considering both the density and the modularity, LF-PIN outperforms other protein complexes detection methods that only consider density or modularity, especially in identifying known protein complexes with low density or low modularity.

## Background

Identifying protein complex is important in understanding the cellular organizations and functional mechanisms. However, the experimental methods to discover protein complexes are costly and time-consuming. Fortunately, with the development of high-throughput techniques such as yeast-two-hybrid [1], mass spectrometry [2], and protein chip technologies [3], protein-protein interactions (PPIs) are increasing fast and available conveniently, which results that large PPI networks for various species can be downloaded easily from public biological databases such as DIP [4], MIPS [5] and SGD [6]. Furthermore, many evidences have indicated that PPI network is a "small-world" network [7,8]. Cliques and dense subgraphs in it generally correspond to the protein complex [9-13]. Thus, a series of protein complexes detection methods are emerged based on mining dense subgraphs in PPI network and namely density-based methods [14-23].

Density-based methods, such as CPM [15,16], CP-DR [17], CMC [18], DPClus [19], IPCA [20], SPICi [21], and Core-Attachment [22], identify protein complexes in PPI networks based on detecting cliques or dense subgraphs. For example, CPM proposes a clique percolation method to mine adjacent k-cliques chains as protein complexes. CP-DR modified CPM by adding distance restriction. CMC algorithm first generates a weighted PPI network by an iterative scoring method and then identifies protein complexes by removing or merging highly overlapped maximal cliques of this weighted PPI network based on their interconnectivity. DPClus, IPCA and SPICi are all "seed-expanding" methods, which identify protein complexes by expanding seeds to density clusters by recursively adding the qualifying neighbours. Core-Attachment algorithm first mines complex core as dense subgraph and then identifies protein complex with its core and attachments separately.

The density-based methods can identify known protein complexes with high density effectively, but they will ignore the protein complexes with low density. However, many protein complexes are not dense subgraphs. For example, out of the 408 known protein complexes of *S.cerevisiae *which are provided by Pu S *et al.* in [24], 89 complexes have density lower than 0.5. Moreover, when they mining protein complexes, most of these methods may neglect many peripheral proteins that connect to the core protein clusters with few links, even though these peripheral proteins are also very important to the protein complex.

To solve this problem, many researchers investigated topologies of protein complexes in PPI networks and found that many protein complexes are densely connected within themselves but sparsely connected with the rest of the PPI network [9-13]. Thus, Radicchi *et al. *[25] proposed in-degree and out-degree of nodes in a subgraph to describe the connections within the subgraph and the connections of the subgraph with the rest of the graph. Radicchi *et al. *[25] and Li *et al. *[26,27] considered the modularity of a subgraph as the sum of the in-degree of all its vertices, divided by the sum of the out-degree of all its vertices. Radicchi *et al. *defined the weak module as a subgraph whose modularity is more than 1, and Luo *et al. *[13] proposed a hierarchical clustering algorithm, Monet, to identify protein complexes as weak modules. Li *et al. *defined the *λ*-module as a subgraph whose modularity is more than the given *λ *value, and proposed a fast hierarchical clustering algorithm, FAG-EC, to identify protein complexes as *λ*-module. Wang *et al.*[28] modified FAG-EC and proposed HC-PIN algorithm to identify protein complexes in weighted PPI network. Based on *λ*-module, Ren *et al. *[29] proposed MOMA algorithm and Wang *et al.*[30] proposed OH-PIN algorithm to identify overlapping and hierarchical protein complexes in PPI network. Lancichinetti *et al.*. [31] defined a fitness function *f *of a subgraph as the sum of the in-degree of all its vertices, divided by the sum of the degree (the sum of in-degree and out-degree) of all its vertices, and proposed NFC algorithm to identify protein complexes as subgraphs with the local maximum fitness value in PPI network. Obviously, a subgraph's fitness value has positive correlation with its modularity. Wang *et al.*[32] modified NFC algorithm by using essential proteins as seeds and proposed EPOF algorithm.

All the above algorithms identify protein complexes as subgraphs in PPI network with high modularity. So they are considered as modularity-based methods. These modularity-based methods can identify protein complexes with different densities, but they usually ignore protein complexes with low modularity. However, many protein complexes are dense subgraphs with low modularity. For example, out of the 408 known protein complexes of *S.cerevisiae*, 254 complexes have *λ *value (modularity) lower than 0.5.

Conclusion above, both density-based methods and modularity-based methods have limitation. Density-based methods may neglect protein complexes with low density and modularity-based methods may neglect protein complexes with low modularity. To identify both of these two kinds of protein complexes, dense subgraphs and subgraphs with high modularity, we define the subgraph's fitness by considering both the density and the modularity of a subgraph, and propose a novel algorithm, named LF_PIN, to identify protein complexes by extending each seed edge to a subgraph until its fitness reaches the local maximum value. LF_PIN chooses seed edges according to the edge clustering value because we find that the higher clustering value a PPI has, the more likely it is to be in a protein complex. The experimental results of *S.cerevisiae *and *E.coli *show that LF_PIN outperforms the other competing algorithms in terms of matching with known protein complexes and functional enrichment. Moreover, it can identify known protein complexes with low density or low modularity effectively.

## Methods

### Density, modularity and fitness

Dense subgraphs and modules in PPI network generally correspond to protein complexes. As dense subgraphs may have low modularity and modules may have low density, protein complexes have various values of modularity and density in PPI network. So we need to define a criterion to predict protein complexes with different topology, including those with low density but high modularity and low modularity but high density. To do it, we define a subgraph's fitness by considering both density and modularity and propose a novel protein complex model as a subgraph with the local maximum fitness value in PPI network.

A weighted PPI network is considered as an undirected weighted graph *G *= (*V,E,W*), where each vertex *v*∈*V *represents a protein, each edge <*u,v*>∈*E *represents an interaction between protein *u *and *v*, and each weight *w_u,v_*∈*W *represents the weight of an interaction between protein *u *and *v*. For an undirected weighted graph *G*, the density of a subgraph *H *(*H*⊆ *G*), donated as *q_H _*, is defined as:

(1) $$q_{H}=2*m_{H}/\;)\left(n_{H}*\left(n_{H}-1\right)\right)$$

where *m_H _*and *n_H _*are the number of edges and vertices in *H *respectively.

For a vertex *v *in a subgraph *H *of an undirected weighted graph *G*, its weighted in-degree, denoted as $d_{w}^{in}\left(H,v\right)$, is the sum of weights of edges connecting vertex *v *to other vertices in *H*; its weighted out-degree, denoted as $d_{w}^{out}\left(H,v\right)$, is the sum of weights of edges connecting vertex *v *to other vertices in *G-H*; and its weighted degree, denoted as *d_w_*(*H,v*), is the sum of its weighted in-degree and its weighted out-degree [28,32].

(2) $$d_{w}^{in}\left(H,v\right)=\underset{u,v\in H,w_{u,v}\in W}{\sum }w_{u,v}$$

(3) $$d_{w}^{out}\left(H,v\right)=\underset{v\in H,u\notin H,w_{u,v}\in W}{\sum }w_{u,v}$$

(4) $$d_{w}\left(H,v\right)=d_{w}^{in}\left(H,v\right)+d_{w}^{out}\left(H,v\right)$$

Generally, the subgraph's modularity is defined as the sum of in-degree of all its vertices, divided by the sum of out-degree of all its vertices [25-30,33]. Obviously, this modularity takes value from 0 to ∞. To make the value range of modularity is as same as that of density, we refer to the fitness function of NFC and EPOF and modify the subgraph's modularity in an undirected weighted graph as the sum of weighted in-degree of all its vertices, divided by the sum of weighted degree of all its vertices [31,32].

(5) $$md_{H}=\underset{v\in H}{\sum }d_{w}^{in}\left(H,v\right)/\;)\underset{v\in H}{\sum }d_{w}\left(H,v\right)$$

Obviously, *md_H _*takes value from 0 to 1. If a subgraph has higher modularity, it has more connections within itself and less connection to the rest of the PPI network. When a subgraph's modularity is equal to 1, it has no connection to the rest of the PPI network.

By considering both subgraph's density and modularity, the fitness of a subgraph *H *in an undirected weighted graph *G*, denoted as *f_ρ_*(*H*), is defined as

(6) $$f_{\rho }\left(H\right)={q_{H}}^{\rho }*m{d_{H}}^{1-\rho }$$

where the parameter ρ decides the importance of density in the fitness and takes value from 0 to 1.

Based on the above definition of fitness, the fitness of a vertex *v *with respect to a subgraph *H*, denoted as *f_ρ_*(*v,H*), is defined as the difference of the fitness of the subgraph *H *with and without vertex *v *[31-33].

(7) $$f_{\rho }\left(v,H\right)=f_{\rho }\left(H+\left\{v\right\}\right)-f_{\rho }\left(H-\left\{v\right\}\right)$$

where *f_ρ_*(*H+*{*v*}) is the fitness of the subgraph in which vertex *v *is added to *H *and *f_ρ_*(*H-*{*v*}) is the fitness of the subgraph in which vertex with *v *is removed from *H*.

When subgraph is a singleton edge, it has the maximum density of 1. When subgraph is the whole graph, it has the maximum modularity of 1. Generally, with the expanding of a subgraph, its modularity is increasing and its density is decreasing. Thus, by expanding from an edge, we can obtain a subgraph with the local maximum fitness value and output it as a complex. The process of a complex extending from an edge is adding neighbor vertices into the subgraph or removing vertices from the subgraph when the inclusion of a new neighbor vertex or the elimination of one vertex from the subgraph will increase the subgraph fitness.

### Seed selecting

How to select the seeds is very important for identifying protein complexes. Obviously, the seeds are edges which have more possibility to be in protein complexes. Moreover, it is obvious that comparing with applying to an un-weighted PPI network, the performance of a protein complex method can be improved when applying to a weighted PPI network whose edge's weight reflects the possibility of the edge in a protein complex [34-36]. So, if the input PPI network is a weighted PPI network and its edge's weight represents the possibility of the edge to be in a protein complex, seed are simply chosen as those edges with weight more than average weight. If the input PPI network is an un-weighted PPI network or a weighted PPI network but its edge's weight cannot reflect the possibility of the edge to be in a protein complex (for example, the weight represents the PPI's confidence), a weighted PPI network should be constructed from the input PPI network and its edge's weight represents the possibility of the edge to be in a protein complex. Then, our method LF-PIN can be applied to this weighted PPI network.

Wang *et al.*[28] defined the clustering value of an edge in a weighted and an un-weighted graph, and pointed out that two vertices connected by an edge with larger clustering value are more likely to lie in the same module. As modules in PPI network generally correspond to protein complexes [9-13], a PPI with higher edge clustering value in a PPI network has more possibility to be in a protein complex. So, it is reasonable to build a weighted PPI network by calculating its edge's weight according to the edge's clustering value and apply LF-PIN to this weighted PPI network. The clustering value of an edge <*u,v*> in a graph *G*, donated as *ECV*(*u,v*), is defined as [28]:

(8) $$ECV\left(u,v\right)=\frac{\sum_{k\in I_{u,v}}w_{u,k}*\sum_{k\in I_{u,v}}w_{v,k}}{\sum_{s\in N_{u}}w_{u,s}*\sum_{t\in N_{v}}w_{v,t}}|$$

where *w_u,k _*is the weight of edge <*u,k*> when *G *is a weighted graph and is equal to 1 when *G *is an un-weighted graph, the *N_u_* and *N_v_* are the sets of neighbors of vertex *u *and vertex *v *respectively, and *I_u,v _*denotes the set of common vertices in *N_u _*and *N_v _*(i.e., *I_u,v _*= *N_u _*∩*N_v_*).

However, in a PPI network, there are many edges whose clustering values are equal to 0. It is obvious that these edges cannot be deleted from the PPI network. As they also have little possibility to be in protein complexes, their weights are set as a small constant, which reflects their small possibility in protein complexes. Thus, the weight of an edge <*u,v*> in a PPI network *G *is calculated as:

(9) $$w\left(u,v\right)=\alpha +\frac{1-\alpha }{ECV_{avg}}*ECV\left(u,v\right)$$

where *α *is the weight of an edge with *ECV *= 0, *ECV_avg _*is the average clustering value of all edges in *G*, including the edges with *ECV *= 0. *α *is set as a constant whose value is much smaller than 1 because the average weight is equal to 1 and the possibility of an edge whose *ECV *= 0 to be in a protein complex is much less than that of an edge selected randomly. For example, out of 15166 PPIs in the PPI network of *S.cerevisiae *download from DIP database [37], 2130 PPIs (14%) are in protein complexes. Out of 8573 PPIs whose *ECV *= 0 in the PPI network, 231 PPIs (2.7%) are in protein complexes. The possibility of a PPI whose *ECV *= 0 to be in a protein complex is only one fifth of that of a PPI selected randomly. So, for this PPI network, the value of *α *is set as 0.2.

### Algorithm LF-PIN

**Figure 1:**
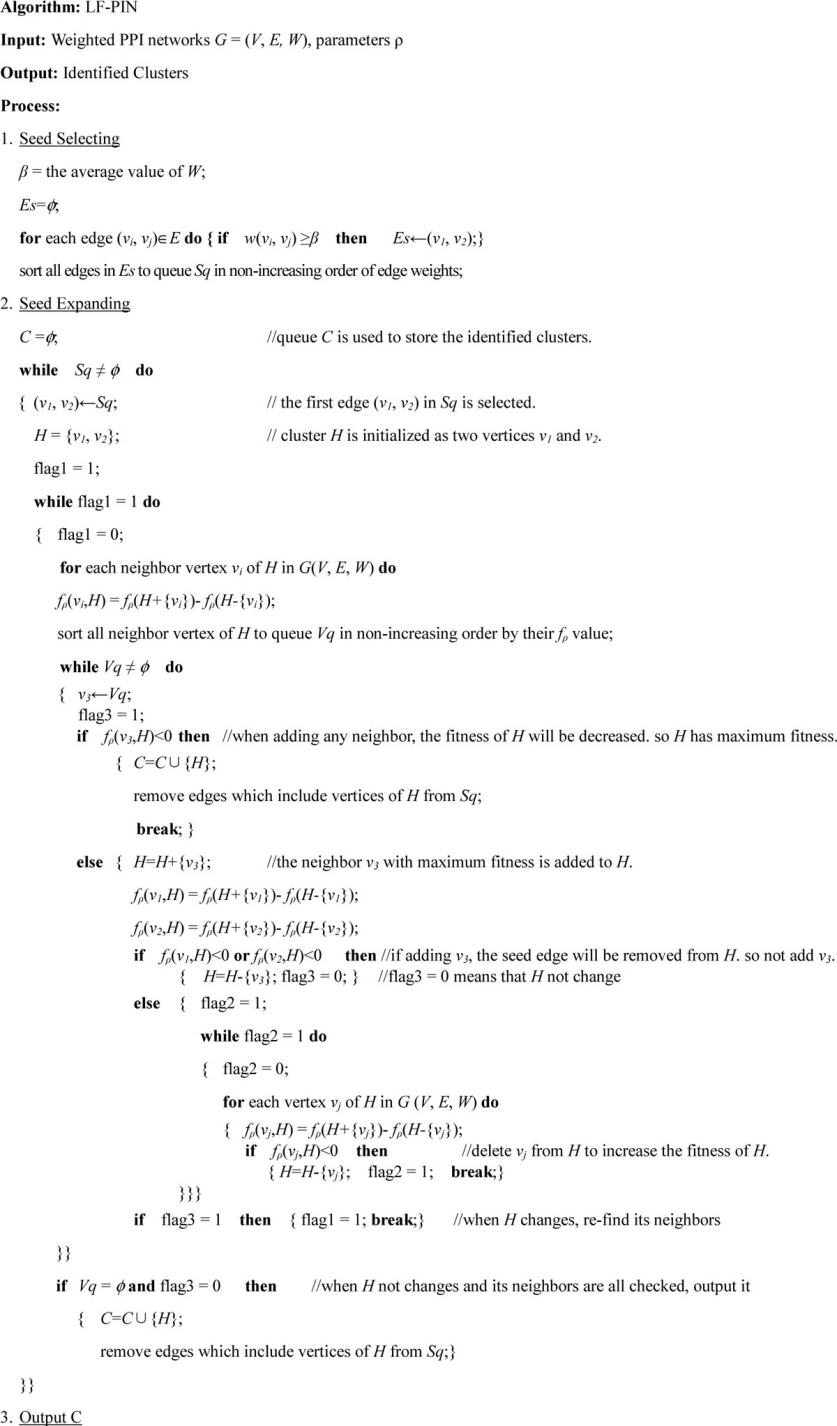
**Description of algorithm LF-PIN**. The figure shows the pseudocode of our method LF-PIN

Based on quantitative description of protein complexes, we propose a novel clustering algorithm LF-PIN (based on **L**ocal **F**itness) to identify protein complex in a weighted PPI network whose edge's weight reflects the possibility of the edge to be in a protein complex. The detailed description of algorithm LF-PIN is shown in Figure 1. The input of algorithm LF-PIN is parameters ρ, and a weighted PPI network which is described as a simple undirected graph *G(V, E, W)*. Algorithm LF-PIN has three stages: seed selecting, seed expanding, and outputting. Firstly, seed are selected as edges whose weights no less than average weight and sorted into seed queue *Sq *in non-increasing order by the edge weight. Then, when the seed queue *Sq *is not null, LF-PIN will always select the first edge in *Sq *as the seed and gradually add neighbor vertex or remove vertex decided by the measure of vertex fitness. If the expanding cluster has neighbor vertices with fitness more than zero, the neighbor vertex with maximum fitness is added to it to increase its fitness. Then, the fitness values of all vertices in the new cluster are recalculated and the vertices whose fitness are negative are deleted. However, it is possible that when adding a neighbor vertex, the seed's vertex will have negative fitness to the new cluster and will be removed. To avoid this case, if adding a neighbor vertex will results the seed's vertex have negative fitness, this neighbor vertex is discarded and the next neighbor vertex with maximum fitness is checked. The expanding will stop when all neighbor vertices are checked or have negative fitness, and an identified cluster is produced. At the same time, all edges which include vertices in the identified cluster are removed from *Sq*. The seed expanding processes will stop when the seed queue *Sq *is null. At last, LF-PIN outputs all identified clusters.

**Figure 2:**
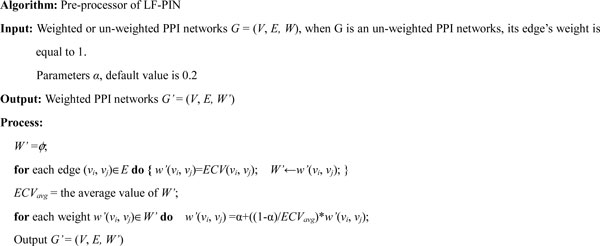
**Description of the pre-processor of LF-PIN**. The figure shows the pseudocode of the pre-processor of LF-PIN

The input PPI network of LF-PIN should be a weighted PPI network whose edge's weight reflects the possibility of the edge to be in a protein complex. If the given PPI network is not a weighted PPI network of this kind, a pre-processor is run to generate the input PPI network from the given PPI network by the method proposed in the above section. The detailed description of the pre-processor is shown in Figure 2. Firstly, each edge's clustering value of the given PPI network is calculated by the formula (8). Then, each edge's weight is calculated according to their clustering value by the formula (9) and the weighted PPI network is generated and output.

## Results and discussion

To evaluate the performance of algorithm LF-PIN, we compare it with seven previous competing algorithms: CMC[18], Core-Attachment[22], CPM[15,16], DPClus[19], HC-PIN[28], NFC[31], and MCL[38,39]. The first four algorithms are density-based methods. HC-PIN and NFC are modularity-based methods. MCL is a fast and highly scalable cluster algorithm for PPI networks based on stochastic flow. Obviously, HC-PIN, MCL, and NFC can all identify protein complexes with different density. The values of the parameters in each algorithm are selected from those recommended by the authors and listed in Table 1.

**Table 1:** Parameters of protein complex detection methods used in the paper

Algorithms	Parameter settings
LF-PIN	*α *= 0.2, ρ = 0.2
CMC	*AdjstCD *= 1, *overlap_thres *= 0.5, *merge_thres *= 0.15
Core-Attachment	
CPM	*k *= 3
DPClus	*CP_in_*= 0.5, *D_in_*= 0.9 in *S.cerevisiae *and 0.6 in *E.coli*
HC-PIN	*λ *= 0.5, size = 2
MCL	*inflation *= 2.0
NFC	*α *= 1

The table shows the values of the parameters in each of eight protein complex detection methods we used in the paper. They are selected from those recommended by the authors. Core-Attachment algorithm has no parameter, so the row of 'Core-Attachment' is empty. The value of parameter *D_in _*of DPClus is different in *S.cerevisiae *and *E.coli*. The values of the parameters of other algorithms are same in *S.cerevisiae *and *E.coli*.

The original un-weighted PPI networks of *S.cerevisiae *and *E.coli *are downloaded from DIP database [37] updated to Jun. 14, 2010 and Oct. 10, 2010, respectively. To generate the input PPI network of LF-PIN, we first removed all self-connecting interactions and repeated interactions, then change the DIP ID of all proteins to ORFname or UniProtKB ID by tool ID Mapping (http://www.uniprot.org/mapping/), finally generate the input PPI network by pre-processor. Here, the value of *α *is set as 0.2. The final input network of *S.cerevisiae *includes 4,746 proteins and 15,166 interactions, which is listed in the Additional file 1. The final input network of *E.coli *includes 2,727 proteins and 11,803 interactions, which is listed in the Additional file 2.

In the section, evaluation methods used in our experiments are described firstly. Secondly, the effect of parameter ρ on clustering results is discussed. Thirdly, all the identified complexes of LF-PIN and those of seven other algorithms in *S.cerevisiae *are compared with the known protein complexes of *S.cerevisiae *in [24], which are listed in the Additional file 3. Fourthly, performances of LF-PIN and seven other algorithms for identifying protein complexes with low density or low modularity are discussed. Finally, all the identified complexes of LF-PIN and those of seven other algorithms in both *S.cerevisiae *and *E.coli *are compared in terms of functional enrichment. The original data of figures in the section are listed in the Additional file 4.

### Evaluation methods

Two criteria are used in the paper to evaluate the performance of protein complex detection methods. One is matching the identified protein complex set with known protein complex set. Another is the functional enrichment of the identified protein complexes.

To determine how effectively a predicted complex (*Pc*) matches a known complex (*Kc*), the overlapping score *OS(Pc, Kc) *between a predicted complex *Pc *and a known complex *Kc *is calculated as [12,19,28]:

(10) $$OS\left(Pc,Kc\right)=|V_{Pc}\cap V_{Kc}{|}^{2}/\;)|V_{Pc}|*|V_{Kc}|$$

where *|V_Pc_*| is the number of proteins in the predicted complex and *|V_Kc_*| is the number of proteins in the known complex. A predicted complex *Pc *and a known complex *Kc *are considered as a match if their overlapping score *OS(Pc, Kc) *is no less than a specific threshold. Generally, the typical value of the threshold is selected as 0.2 [12,19,28]. If *OS(Pc, Kc) *is equal to 1, we say that they are perfectly matched. Based on the matching of known complexes and predicted complexes, three popular evaluation criteria, *Specificity *(*Sp*), *Sensitivity *(*Sn*) and *F-score*, are used to quantify the quality of protein complexes detection methods. *Specificity *is the fraction of the predicted complexes that are matched by the known complexes among all the predicted complexes [12]. *Sensitivity *is the fraction of the known complexes that are matched by the predicted complexes among all the known complexes [12]. *F-score *combines the *Sensitivity *and *Specificity *and is defined as [28]:

(11) $$F-score=2*Sn*Sp/\;)\left(Sn+Sp\right)$$

As *F-score *considers both *Sensitivity *and *Specificity*, it is a comprehensive evaluation and used as prediction accuracy in the paper.

To evaluate the functional enrichment of predicted protein complexes, the P-value of a protein complex with a given GO term is used to estimate whether the proteins in the complex are enriched for the GO term with a statistically significant probability compared to what one would expect by chance[19,40]. The smaller P-value indicates the predicted protein complexes is not accumulated at random and is more biologically significant than the one with a larger P-value [28,41]. As a protein complex has various P-values for various GO terms, its P-value defaults to its minimum P-value.

### Effect of parameter ρ

**Figure 3:**
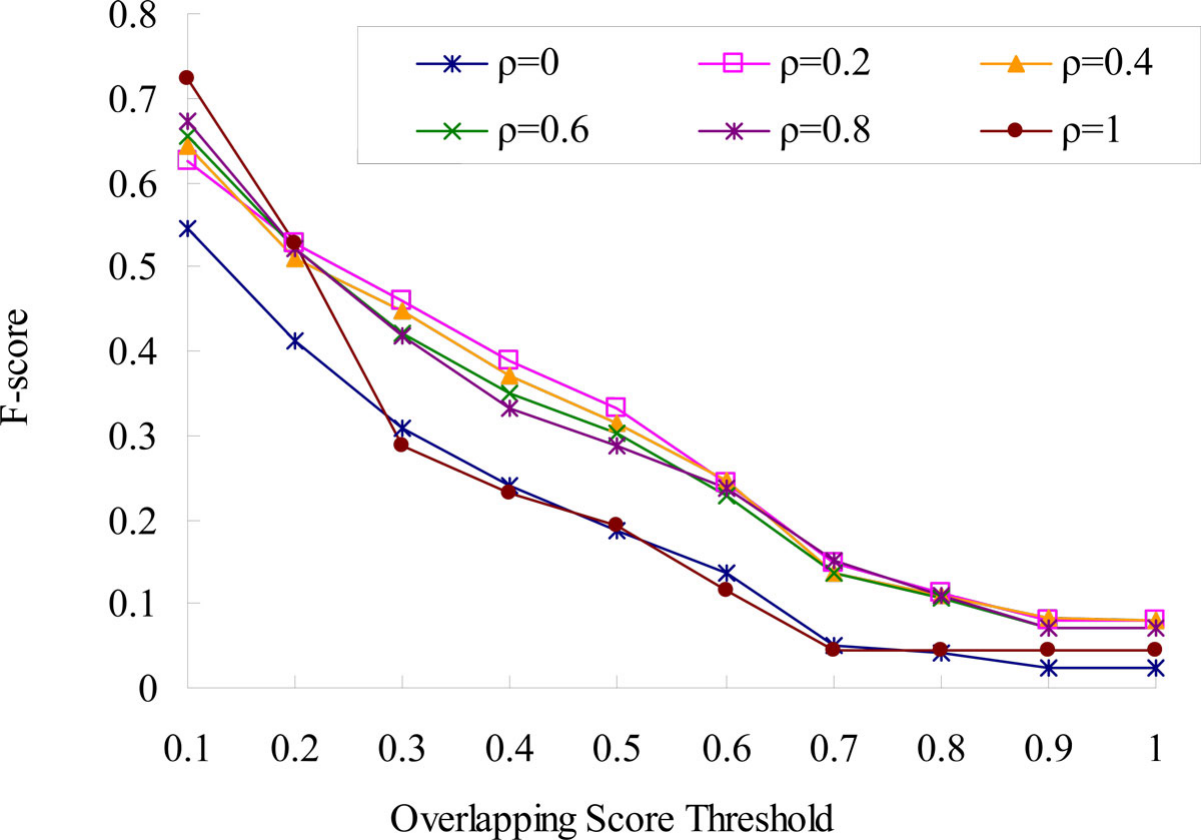
**Effect of parameter ρ on the F-score of LF-PIN (for *S.cerevisiae*)**. The figure shows the six *F-score *curves of LF-PIN with ρ = 0, ρ = 0.2, ρ = 0.4, ρ = 0.6, ρ = 0.8, and ρ = 1 respectively.

The parameter ρ of LF-PIN decides the importance of density in the fitness. It takes value from 0 to 1. To evaluate the effect of the parameter ρ on the clustering results, we change the values of parameter ρ from 1 to 0 with 0.2 decrements and achieve eleven different output sets of protein complexes from the PPI network of *S.cerevisiae*. The experimental results are shown in Table 2 and Figure 3.

**Table 2:** The effect of the variation of ρ on clustering (for *S.cerevisiae*)

ρ	Number	Average Size	Average Density	Minimum Density	Average Modularity	Minimum Modularity
1	792	2.00	1.00	1.00	0.11	0.01
0.8	396	3.60	1.00	0.96	0.24	0.04
0.6	377	3.75	0.99	0.83	0.25	0.04
0.4	349	4.14	0.94	0.52	0.27	0.06
0.2	297	5.10	0.84	0.17	0.31	0.08
0	238	12.52	0.45	0.03	0.44	0.13

The table shows when different values of parameter ρ are selected, the number, the average size, the average and the minimum density, the average and the minimum modularity of the protein complexes identified by LF-PIN.

As shown in Table 2, with the decrease of ρ, the number and the density of identified complexes are decreasing; the size and the modularity of identified complexes are increasing. Generally, with the increase of a subgraph's size, its modularity is increasing and its density is decreasing. Meanwhile, with the decrease of ρ, the density is less important and the modularity is more important in the fitness. So, with the decrease of ρ, a seed edge should be expanded to a larger subgraph with smaller density and larger modularity. As shown in Figure 3, when overlapping score's threshold is no less than 0.2, the F-scores of LF-PIN with ρ = 1 and ρ = 0 are much less than those of LF-PIN with ρ in range from 0.2 to 0.8. As LF-PIN predicts protein complexes considering only subgraph's density when ρ = 1, considering only subgraph's modularity when ρ = 0, and considering both subgraph's density and modularity when 0<ρ<1, the F-score curves in Figure 3 imply that compared with only considering density or modularity, the prediction accuracy can be improved by considering both density and modularity. The F-scores of LF-PIN with ρ in range from 0.2 to 0.8 are very close, which means the performance of LF-PIN with ρ in range from 0.2 to 0.8 are close. As shown in Table 2, when ρ is in the range of 0.4 to 0.8, the density of identified protein complexes are too high (nearly 1). So, to identify various protein complexes, including those with low density and low modularity, the value of parameter ρ is selected as 0.2 in the paper.

### Comparison with known complexes

**Figure 4:**
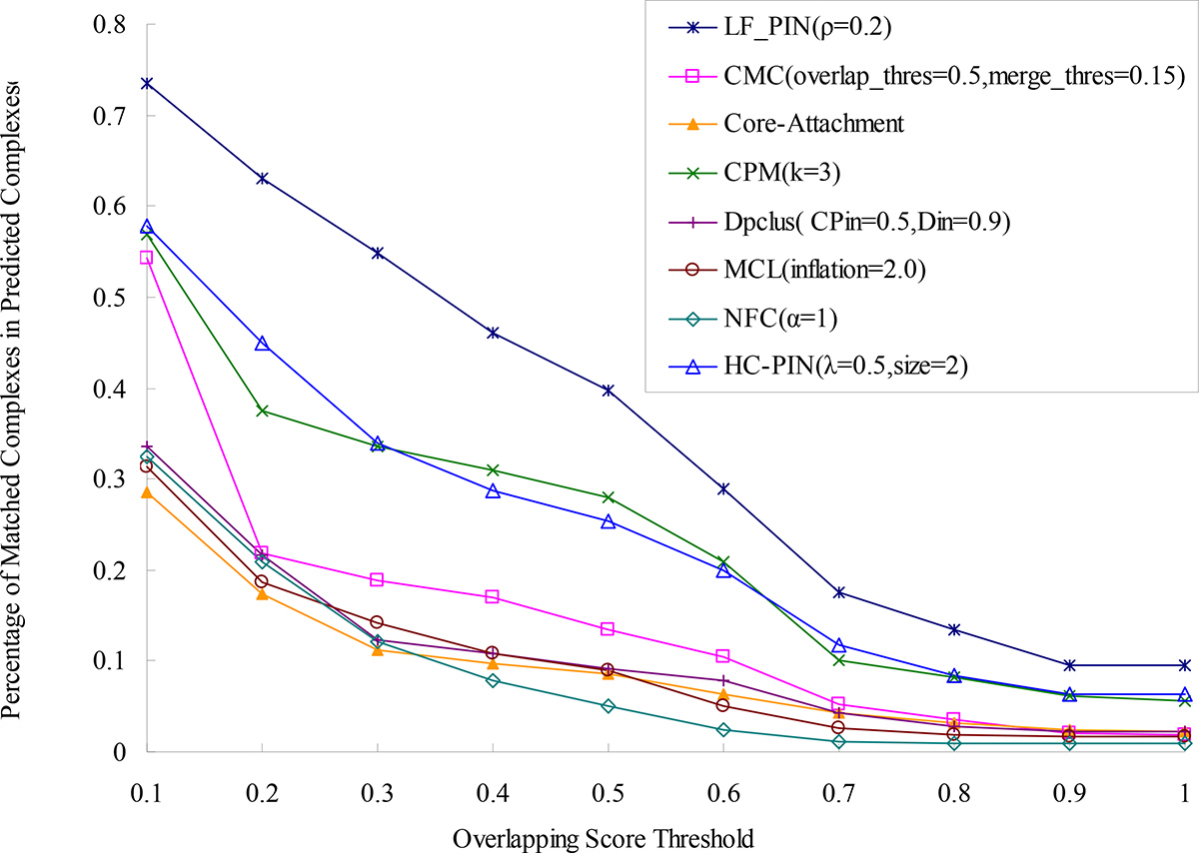
**Comparison of the percentage of matched predicted complexes of LF-PIN and other algorithms (for *S.cerevisiae*)**. The figure shows the percentages of matched predicted complexes of LF-PIN, CMC, Core-Attachment, CPM, DPClus, HC-PIN, MCL and NFC, with respect to different overlapping scores threshold.

To directly validate the effectiveness of algorithm LF-PIN for identifying protein complexes, we compare the protein complexes predicted by LF-PIN and other seven algorithms with the known protein complexes obtained from [24] and list the percentage of matched predicted complexes of these eight algorithms in Figure 4. We can see from Figure 4 that when overlapping score's threshold is equal to 0.2 (the typical value of overlapping score's threshold used in many literature), 63% complexes detected by LF-PIN are matched by the known complexes. This ratio is much higher than those identified by other seven competing algorithms at the same threshold. For example, when overlapping score's threshold is equal to 0.2, 45% complexes predicted by HC-PIN are matched, which is the best result in the seven competing algorithms. Even compared with this best result, 40% improvement can be obtained by using LF-PIN algorithms. Furthermore, Figure 4 shows that for each overlapping score's threshold, the percentage of matched complexes in the complex set identified by LF-PIN is much higher than those identified by other seven competing algorithms. All these indicate that LF-PIN outperforms other seven competing algorithms in terms of matching with the known complexes.

To compare the performance of LF-PIN with those of other seven competing algorithms, we calculate *Sensitivity, Specificity, F-score*, the number and the percentage of perfect matches of the eight algorithms and list them in Table 3. Here, the overlapping score's threshold is selected as 0.2. As shown in Table 3, the number of complexes identified by LF-PIN is 297, which is much less than those identified by CMC (only 26%), Core-Attachment (only 22%), and DPClus (only 25%). Obviously, the more complexes dose an algorithm identify, the more perfect matches and matched complexes dose the algorithm identify. Thus, LF-PIN identifies less perfect matches than Core-Attachment, and its *Sensitivity *value is less than those of CMC, Core-Attachment, and DPClus. However, Table 3 shows that the percentage of perfect matches in the identified complexes and the *Specificity *value of LF-PIN are both higher than those of other seven algorithms, which means that the percentages of perfect matches and matched complexes in the complexes identified by LF-PIN are both higher than those of the other algorithms. Moreover, LF-PIN has the highest *F-score *value in the eight algorithms. Even compared with the highest *F-score *value of other seven algorithms, 41% improvement can be obtained by using LF-PIN algorithms.

**Table 3:** Comparison of Sensitivity, Specificity, F-score, the number and the percentage of perfect matches of LF-PIN and other algorithms (for *S.cerevisiae*)

	Number	Perfect match	Sensitivity	Specificity	F-score
LF-PIN	297	28 (9.43%)	0.452	0.630	0.526
CMC	1130	21 (1.86%)	0.576	0.219	0.317
Core-Attachment	1358	31 (2.28%)	0.589	0.174	0.268
CPM	197	11 (5.58%)	0.185	0.376	0.248
DPClus	1200	27 (2.25%)	0.651	0.216	0.324
HC-PIN	265	17 (6.42%)	0.318	0.449	0.373
MCL	929	15 (1.61%)	0.450	0.187	0.264
NFC	518	5 (0.97%)	0.277	0.209	0.238

The table shows the values of Sensitivity, Specificity, and F-score, the numbers and the percentages of perfect matches of LF-PIN, CMC, Core-Attachment, CPM, DPClus, HC-PIN, MCL and NFC in PPI network of *S.cerevisiae*. In column of 'Perfect match', the integers out of brackets are the numbers of perfect matches, the percentages in brackets are the percentages of perfect matches.

**Figure 5:**
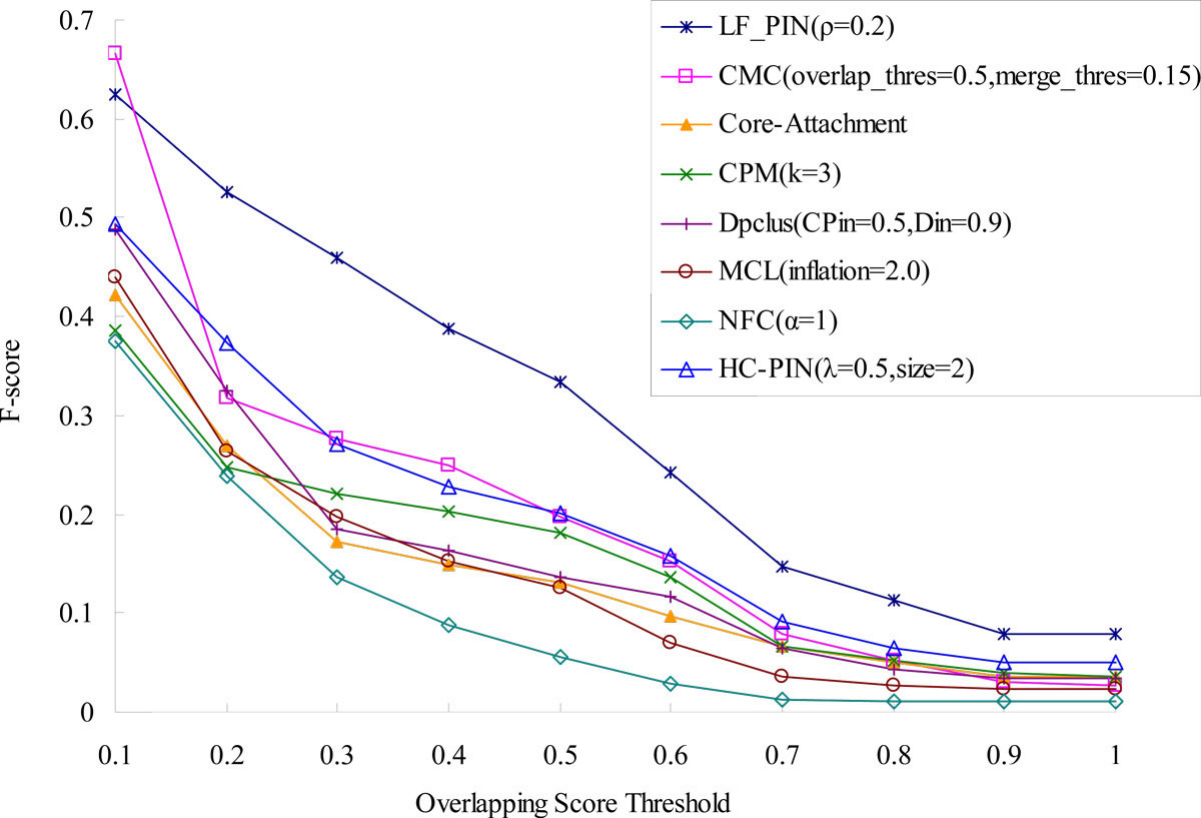
**Comparison of F-score of LF-PIN and other algorithms (for *S.cerevisiae*)**. The figure shows the values of F-score of LF-PIN, CMC, Core-Attachment, CPM, DPClus, HC-PIN, MCL and NFC, with respect to different overlapping scores threshold.

To give a more complete comparison, we compare the *F-score *of LF-PIN and other seven algorithms with respect to different overlapping score's thresholds in Figure 5. As shown in Figure 5, LF-PIN algorithm has the highest value of *F-score *in the eight algorithms when overlapping score's threshold is no less than 0.2, which means it has the highest prediction accuracy in the eight algorithms.

### Comparison with known complexes of low density

**Figure 6:**
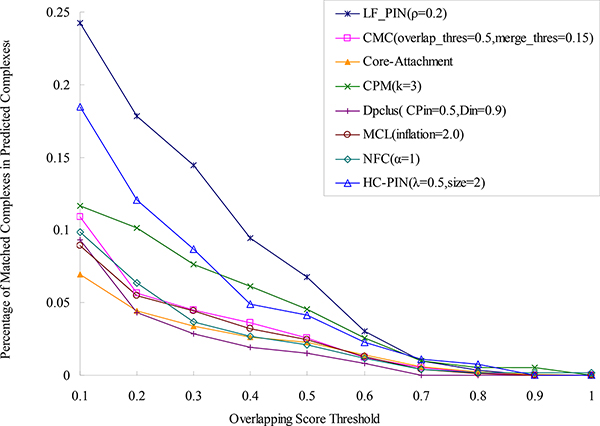
**Comparison of the percentage of matched predicted complexes of LF-PIN and other algorithms**. (Known protein complexes are known protein complexes of *S.cerevisiae *with density less than 0.5). The figure is used to compare the performance of LF-PIN and other algorithms for identifying protein complexes with low density. It shows the percentages of matched predicted complexes of LF-PIN, CMC, Core-Attachment, CPM, DPClus, HC-PIN, MCL and NFC, with respect to different overlapping scores threshold. The known protein complexes are known protein complexes of *S.cerevisiae *with density less than 0.5.

**Figure 7:**
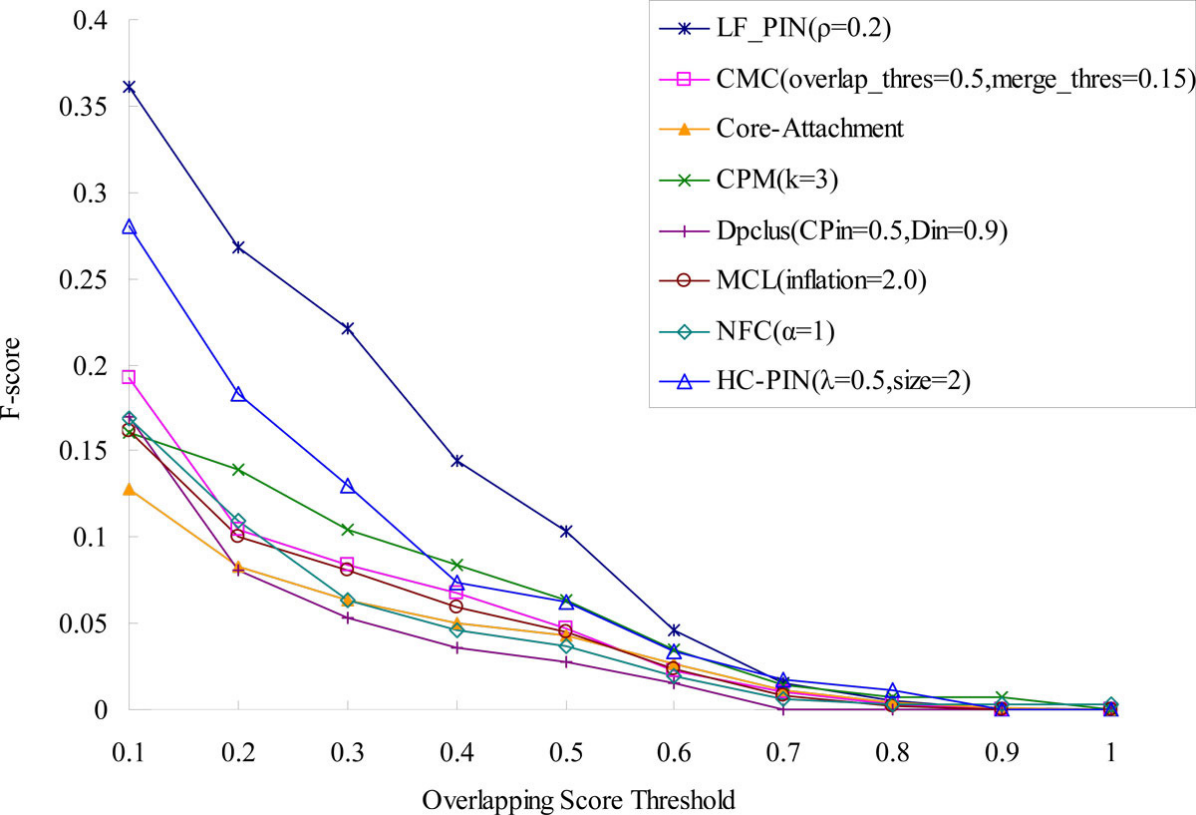
**Comparison of the F-score of LF-PIN and other algorithms**. (Known protein complexes are known protein complexes of *S.cerevisiae *with density less than 0.5). The figure is used to compare the prediction accuracy of LF-PIN and other algorithms for identifying protein complexes with low density. It shows the values of F-score of LF-PIN, CMC, Core-Attachment, CPM, DPClus, HC-PIN, MCL and NFC, with respect to different overlapping scores threshold. The known protein complexes are known protein complexes of *S.cerevisiae *with density less than 0.5.

A more attractive characteristic of LF-PIN is that this algorithm can identify significant protein complexes with low density. We can see from Table 2 that the density of protein complexes identified by LF-PIN (ρ = 0.2) can be as low as 0.17. To directly validate the effectiveness of algorithm LF-PIN for identifying protein complexes with low density, we select all known protein complexes obtained from [24] with density less than 0.5, counting up to 89 complexes, and compare them with the protein complexes predicted by LF-PIN and other seven algorithms in Figure 6 and Figure 7. Figure 6 shows the percentage of matched predicted complexes of LF-PIN and other algorithms. Figure 7 shows the *F-score *of LF-PIN and other algorithms.

We can see from Figure 6 that for each overlapping score's threshold, especially when overlapping score's threshold less than 0.6, the percentage of matched complexes in the complex set identified by LF-PIN is much higher than those identified by other seven algorithms, especially by the four density-based algorithms: CMC, Core-Attachment, CPM and DPClus. For example, when overlapping score's threshold is equal to 0.2, 18% complexes detected by LF-PIN are matched with known complexes with low density. This matched percentage is 3.2 times as that detected by CMC, 4.0 times as that detected by Core-Attachment, 1.8 times as that detected by CPM, and 4.1 times as that detected by DPClus.

As shown in Figure 7, for each overlapping score's threshold, especially when overlapping score's threshold is less than 0.7, the *F-score *of LF-PIN is much higher than those of other seven algorithms, especially for the four density-based algorithms. For example, when overlapping score's threshold is equal to 0.2, the *F-score *of LF-PIN is 0.268, which is 2.6 times as that detected by CMC, 3.2 times as that detected by Core-Attachment, 1.9 times as that detected by CPM, and 3.3 times as that detected by DPClus.

Conclusion above, compared with other seven algorithms, especially for the four density-based algorithms, LF-PIN has much better performance for identifying protein complexes with low density. When overlapping score's threshold is set to 0.2, compared with the four density-based algorithms, the prediction accuracy can be improved no less than 90% by using LF-PIN algorithm.

### Comparison with known complexes of low modularity

**Figure 8:**
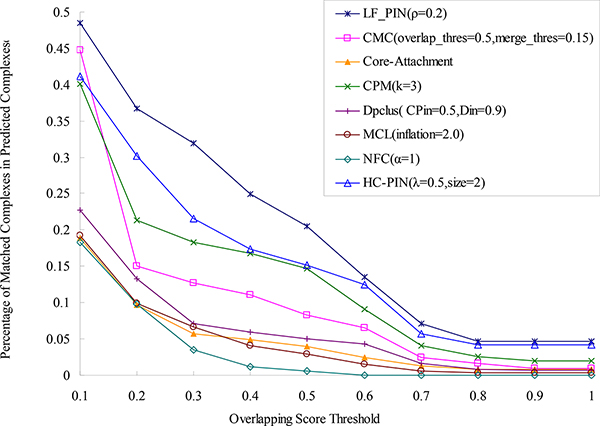
**Comparison of the percentage of matched predicted complexes of LF-PIN and other algorithms**. (Known protein complexes are known protein complexes of *S.cerevisiae *with modularity less than 0.3). The figure is used to compare the performance of LF-PIN and other algorithms for identifying protein complexes with low modularity. It shows the percentages of matched predicted complexes of LF-PIN, CMC, Core-Attachment, CPM, DPClus, HC-PIN, MCL and NFC, with respect to different overlapping scores threshold. The known protein complexes are known protein complexes of *S.cerevisiae *with modularity less than 0.3.

**Figure 9:**
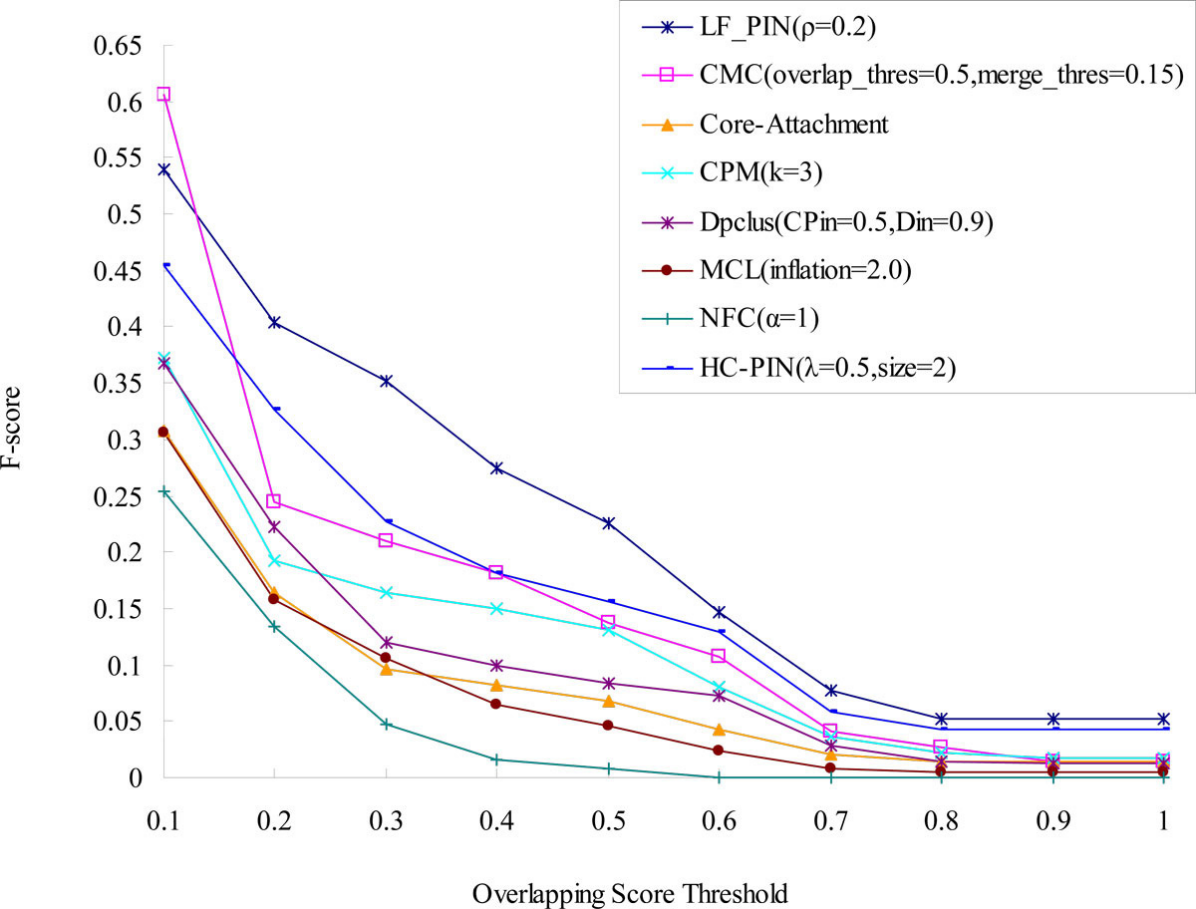
**Comparison of the F-score of LF-PIN and other algorithms**. (Known protein complexes are known protein complexes of *S.cerevisiae *with modularity less than 0.3). The figure is used to compare the prediction accuracy of LF-PIN and other algorithms for identifying protein complexes with low modularity. It shows the values of F-score of LF-PIN, CMC, Core-Attachment, CPM, DPClus, HC-PIN, MCL and NFC, with respect to different overlapping scores threshold. The known protein complexes are known protein complexes of *S.cerevisiae *with modularity less than 0.3.

Another attractive characteristic of LF-PIN is that this algorithm can identify significant protein complexes with low modularity. We can see from Table 2 that the modularity of protein complexes identified by LF-PIN (ρ = 0.2) can be as low as 0.08. To directly validate the effectiveness of algorithm LF-PIN for identifying protein complexes with low modularity, we select all known protein complexes obtained from [24] with modularity less than 0.3, counting up to 247 complexes, and compare them with the protein complexes predicted by LF-PIN and other seven algorithms in Figure 8 and Figure 9. Figure 8 shows the percentage of matched predicted complexes of LF-PIN and other seven algorithms. Figure 9 shows the *F-score *of LF-PIN and other seven algorithms.

We can see from Figure 8 that for each overlapping score's threshold, the percentage of matched complexes in the complex set identified by LF-PIN is higher than those identified by other seven algorithms, especially by NFC. For example, when overlapping score's threshold is equal to 0.2, 37% complexes detected by LF-PIN are matched. However, only 10% complexes detected by NFC are matched. The improvement of LF-PIN algorithms is about 2.7 times. Meanwhile, Figure 9 shows that when overlapping score's threshold no less than 0.2, the *F-score *of LF-PIN is also higher than those of other seven algorithms, especially of NFC. For example, when overlapping score's threshold is equal to 0.2, the *F-score *of LF-PIN is 0.404, and the *F-score *of NFC is only 0.135. The *F-score *of LF-PIN is three times as that of NFC.. All these imply that compared with other seven algorithms, especially for the modularity-based algorithm NFC,, LF-PIN has better performance for identifying protein complexes with low modularity.

However, from Figure 8 and Figure 9, we can see that compared with the improvements of using LF-PIN instead of NFC, the improvements of using LF-PIN instead of HC-PIN (another module-based algorithm) is little. For example, when overlapping score's threshold is equal to 0.2, compared with the results of HC-PIN, the percentages of matched complexes identified by LF-PIN is only improved 22% and the *F-score *of LF-PIN is only improved 24%. The possible reason is that the λ value of HC-PIN is only selected as 0.5 in the paper, which result the modularity of the protein complex identified by HC-PIN is smaller (when a subgraph's λ value is 0.5, its modularity is only 0.33). So, the protein complexes identified by HC-PIN can also match well with the known protein complexes with low modularity.

### Comparison with other algorithms in terms of functional enrichment

To evaluate the effectiveness of LF-PIN, we apply it and other seven algorithms in PPI networks of *S.cerevisiae *and *E.coli*, and compare the functional enrichment of protein complexes identified by each algorithm in Table 4 and Table 5, respectively. Here, the GO annotation is downloaded from GO database [42] and Biological Processes are used.

**Table 4:** Comparison of the functional enrichment of protein complexes identified by LF-PIN and other algorithms (for *S.cerevisiae*)

Algorithms	<E-10	[E-10,E-5]	[E-5, 0.01]	≥0.01 insignificant	<0.01 significant
LF-PIN	63(21.2%)	93(31.3%)	103(34.7%)	38(12.8%)	259(87.2%)
CMC	73(6.5%)	191(16.9%)	292(25.9%)	574(50.8%)	556(49.2%)
Core-Attachment	76(5.6%)	122(9.0%)	287(21.1%)	873(64.3%)	485(35.7%)
CPM	25(12.7%)	49(24.9%)	42(21.3%)	81(41.1%)	116(58.9%)
DPClus	42(3.5%)	155(12.9%)	329(27.4%)	674(56.2%)	526(43.8%)
HC-PIN	40(15.1%)	42(15.9%)	84(31.7%)	99(37.4%)	166(62.6%)
MCL	54(5.8%)	114(12.3%)	239(25.7%)	522(56.2%)	407(43.8%)
NFC	63(21.2%)	81(15.6%)	124(23.9%)	266(51.3%)	259(87.2%)

The table lists the percentages of protein complexes identified by LF-PIN, CMC, Core-Attachment, CPM, DPClus, HC-PIN, MCL and NFC in PPI network of *S.cerevisiae *whose P-value falls within *<*E-10, [E-10, E-5], [E-5, 0.01] and ≥0.01.

**Table 5:** Comparison of the functional enrichment of protein complexes identified by LF-PIN and other algorithms (for *E*.coli)

Algorithms	[E-10,E-5]	[E-5, 0.01]	≥0.01 insignificant	<0.01 significant
LF-PIN	11(7.6%)	23(16.0%)	110(76.4%)	34(23.6%)
CMC	13(2.9%)	46(10.2%)	391(86.9%)	59(13.1%)
CPM	4(6.3%)	6(9.5%)	53(84.1%)	10(15.9%)
DPClus	11(7.6%)	51(10.8%)	410(87.0%)	61(13.0%)
HC-PIN	6(2.1%)	12(8.3%)	122(84.1%)	23(15.9%)
MCL	7(1.2%)	51(8.9%)	515(89.9%)	58(10.1%)
NFC	10(2.1%)	41(14.3%)	240(83.6%)	47(16.4%)

The table lists the percentages of protein complexes identified by LF-PIN, CMC, CPM, DPClus, HC-PIN, MCL and NFC in PPI network of *E.coli *whose P-value falls within [E-10, E-5], [E-5, 0.01] and ≥0.01.

As shown in Table 4, only 12.8% protein complexes identified by LF-PIN are insignificant (Generally, a complex with P-value≥0.01 is considered insignificant and that with P-value<0.01 is considered significant). This percentage is much lower than those of other seven algorithms. For example, the percentage of insignificant complexes identified by HC-PIN is 37.4%, which is the lowest in the seven other algorithms. Even this lowest percentage is about 3 times as that identified by LF-PIN. On the contrary, the percentage of significant complexes identified by LF-PIN, including those of complexes whose P-value falls within *<*E-10, [E-10, E-5], [E-5, 0.01], are all higher than those identified by other seven algorithms. All these mean that LF-PIN is more effective for identifying significant proteins complexes than other algorithms in *S.cerevisiae*.

From Table 5, we can draw the same conclusion in *E.coli*. The percentage of insignificant complexes identified by LF-PIN is lower than those identified by other seven algorithms. On the contrary, compared with the results of other seven algorithms, the percentage of significant complexes identified by LF-PIN is improved 44% to 133%. The statistical results of Table 4 and Table 5 indicate that LF-PIN has good performance for identifying significant proteins complexes.

## Conclusions

In the post-genome era, one of the most important works is to discover the protein complexes with various density and modularity. In this paper, we propose a novel fitness function by considering both density and modularity and develop a fitness-based local search algorithm, named LF-PIN, to identify protein complexes with different density and modularity in PPI network. By tuning the value of parameter ρ in the fitness function, we can adjust the importance of density and modularity in the fitness. Experimental results in *S.cerevisiae *show that compared with considering only density (ρ = 1) or only modularity (ρ = 0), LF-PIN has better performance when considering both density and modularity (0<ρ<1). To compare algorithm LF-PIN with other protein complexes detection methods, we apply LF-PIN and other seven competing algorithms, including CMC, Core-Attachment, CPM, DPClus, HC-PIN, MCL, and NFC, to the protein interaction network of *S.cerevisiae *and *E.coli*. The experimental results in both *S.cerevisiae *and *E.coli *show that LF-PIN identifies much more significant protein complexes and generates much less insignificant protein complexes than other algorithms. When matching with the known protein complexes of *S.cerevisiae*, for each overlapping score's threshold, LF-PIN has the highest percentage of matched predicted complexes and the highest *F-score*. These quantitative comparisons reveal that our algorithm LF-PIN outperforms the other previous competing algorithms in identifying protein complexes. Moreover, algorithm LF-PIN has good performance for identifying protein complexes with low density or low modularity. When matching with the known protein complexes of *S.cerevisiae *with density less than 0.5, the percentage of matched predicted complexes and the *F-score *of LF-PIN are both higher than those of other seven algorithms, especially to the four density-based algorithms, CMC, Core-Attachment, CPM and DPClus. When matching with the known protein complexes of *S.cerevisiae *with modularity less than 0.3, the percentage of matched predicted complexes and the *F-score *of LF-PIN are also both higher than those of other seven algorithms, especially to the modularity-based algorithms NFC.

## Competing interests

The authors declare that they have no competing interests.

## Authors' contributions

JR obtained the protein-protein interaction data, essential proteins and Orthologous data. JR and JXW designed the new method, LF-PIN. JR and ML analyzed the results. JR, JXW and LSW drafted the manuscript together. All authors have read and approved the manuscript.

## Supplementary Material

Additional file 1 The input PPI network of LF-PIN for *S.cerevisiae*. Description: This file provides the input PPI network of LF-PIN for *S.cerevisiae*. It has three columns, 'Protein A', 'Protein B', and 'Weight'. 'Protein A' and 'Protein B' are PPI's two proteins and 'Weight' is PPI's weight. The original un-weighted PPI network of the input PPI network is downloaded from DIP database (version 20100614). To generate the input PPI network, we first removed all self-connecting interactions and repeated interactions, then change the DIP ID of all proteins to ORFname by tool ID Mapping (http://www.uniprot.org/mapping/), finally generate the input PPI network by pre-processor. Here, the value of *α *is set as 0.2. Click here for file

Additional file 2 The input PPI network of LF-PIN for *E.coli*. Description: This file provides the input PPI network of LF-PIN for *E.coli*. It has three columns, 'Protein A', 'Protein B', and 'Weight'. 'Protein A' and 'Protein B' are PPI's two proteins and 'Weight' is PPI's weight. The original un-weighted PPI network of the input PPI network is downloaded from DIP database (version 20101010). To generate the input PPI network, we first removed all self-connecting interactions and repeated interactions, then change the DIP ID of all proteins to UniProtKB ID by tool ID Mapping, finally generate the input PPI network by pre-processor. Here, the value of *α *is set as 0.2. Click here for file

Additional file 3 The known protein complexes of *S.cerevisiae*. Description: This file provides the known protein complexes of *S.cerevisiae *which is obtained in [36]. Click here for file

Additional file 4 The original data of Figure 3 to Figure 9. Description: This file provides the original data of Figure 3 to Figure 9. Click here for file

## Declarations

The publication costs for this article were funded by the corresponding author.

This article has been published as part of *BMC Systems Biology *Volume 7 Supplement 4, 2013: Selected articles from the IEEE International Conference on Bioinformatics and Biomedicine 2012: Systems Biology. The full contents of the supplement are available online at http://www.biomedcentral.com/bmcsystbiol/supplements/7/S4.
